# Integrative Peptide Drug Development: Chemical Engineering, AI-Driven Design, and Cell-Penetrating Peptides

**DOI:** 10.3390/pharmaceutics18050537

**Published:** 2026-04-28

**Authors:** Yong Eun Jang, Minjun Kwon, Chan Woo Kwon, Seok Gi Kim, Ji Su Hwang, Nimisha Pradeep George, Seung Ryong Paik, Sampa Misra, Shaherin Basith, Seung Soo Sheen, Gwang Lee

**Affiliations:** 1 Molecular Science and Technology Research Center, Ajou University, Suwon 16499, Republic of Korea; jye120@ajou.ac.kr (Y.E.J.); js3004@ajou.ac.kr (J.S.H.); 2 Department of Physiology, Ajou University School of Medicine, Suwon 16499, Republic of Korea; kmj936@ajou.ac.kr (M.K.); rlatjrrl9977@ajou.ac.kr (S.G.K.); paiksl0922@gmail.com (S.R.P.); sampamisra@ajou.ac.kr (S.M.); sbasith@ajou.ac.kr (S.B.); 3 Ajou University School of Medicine, Suwon 16499, Republic of Korea; cwkwon99@ajou.ac.kr; 4 Department of Molecular Science and Technology, Ajou University, Suwon 16499, Republic of Korea; nimishapgeorge@ajou.ac.kr; 5 Department of Chemistry, Kyonggi University, Suwon 16227, Republic of Korea; 6 Department of Pulmonary and Critical Care Medicine, Ajou University School of Medicine, Suwon 16499, Republic of Korea

**Keywords:** peptide therapeutics, cell-penetrating peptide, AI drug design, deep learning, machine learning

## Abstract

Peptide therapeutics occupy a unique chemical space between small molecules and biologics, combining high target specificity with structural programmability and favorable safety profiles. Recent regulatory approvals and expanding clinical pipelines underscore the growing therapeutic and commercial relevance of peptide-based drugs. This review outlines chemical modification approaches and contemporary design strategies, and evaluates their impact on proteolytic stability, pharmacokinetics, membrane permeability, and target engagement. We then highlight recent advances in artificial intelligence (AI)-guided peptide drug design, including machine learning models, protein language models, and generative architectures that enable high-throughput activity prediction, property optimization, and de novo sequence generation. These approaches collectively accelerate the traditional discovery–design–validation cycle while reducing experimental attrition through data-driven, structure-informed modeling frameworks. Among these applications, AI also enables the rational design of cell-penetrating peptides (CPPs) to enhance intracellular delivery and biological activity. Building on these methodological advances, we further examine their application to peptide therapeutics, with particular emphasis on AI-based predictive models for CPPs as well as on therapeutic applications within the central nervous and pulmonary systems. We conclude by outlining future perspectives and emphasize that the systematic integration of AI-enabled sequence design with rational chemical engineering and advanced delivery technologies, supported by rigorous experimental validation, will be critical for developing robust and clinically durable peptide-based medicines.

## 1. Introduction

Peptide therapeutics are pharmacologically active agents composed of short chains of amino acids, typically less than 50 residues, designed to modulate specific biological targets [1,2]. Peptide therapeutics have evolved from early breakthroughs such as the clinical introduction of insulin in 1922 [3] and the synthesis of oxytocin and vasopressin by Du Vigneaud et al. [4,5], which together established the foundational paradigm for modern peptide chemistry and chemical biology. These discoveries demonstrated that short polypeptides can mimic complex hormonal activities, thus paving the way for modern peptide drug discovery. The intrinsic biochemical properties of peptides, including specific receptor binding, structural flexibility, and minimal side effects, make them particularly suitable for the development of therapeutic agents targeting diseases such as cancer, human immunodeficiency virus (HIV) infection, chronic pain, osteoporosis, microbial infections, chronic kidney disease, and diabetes [1,6,7]. As of 2025, more than 100 peptide therapeutics have gained global regulatory approval [8], and numerous peptide candidates representing diverse structural classes and mechanisms of action are currently under clinical development [6,9].

Despite an expanding therapeutic portfolio, peptide drugs have long been constrained by intrinsic liabilities, such as metabolic instability, rapid renal clearance, and limited membrane permeability, which have historically hindered their development. These properties often result in short circulation half-lives and poor bioavailability, posing significant challenges for systemic delivery and clinical translation [8,10,11]. To address these challenges, various strategies have been developed, including chemical modification of peptide structures, sequence optimization, and conjugation with functional molecules [11]. Notably, glucagon-like peptide-1 receptor agonists (GLP-1RAs), including liraglutide and semaglutide, have revolutionized the treatment of type 2 diabetes and obesity [12,13]. This underscores how rational chemical modifications, combined with advanced formulation technologies, can effectively overcome the pharmacokinetic barriers typically associated with peptide drugs, leading to extended half-life, improved bioavailability, and enhanced therapeutic efficacy [14].

Alongside chemical optimization, delivery technologies have emerged as a critical approach to improve peptide pharmacokinetics and intracellular accessibility. Notably, cell-penetrating peptides (CPPs) have attracted considerable attention due to their ability to facilitate cellular uptake and enhance intracellular delivery of therapeutic cargos [15,16]. However, the design of effective CPPs remains challenging because cellular uptake efficiency, toxicity, and stability are strongly influenced by peptide sequence and physicochemical properties. As a result, the rational design of peptides, including CPPs, requires systematic exploration of large sequence spaces, posing significant challenges for conventional experimental approaches [17,18].

In parallel with advances in peptide chemistry and formulation technologies, artificial intelligence (AI), including machine learning (ML) and deep learning (DL) approaches, is increasingly transforming peptide drug discovery [19,20,21]. Recent computational frameworks enable large-scale analysis of peptide sequence–structure–function relationships, facilitating the prediction of key properties such as bioactivity, stability, toxicity, and membrane permeability [22,23,24,25,26,27]. Generative models and AI-driven design platforms further enable the de novo generation and optimization of peptide sequences with improved pharmacological profiles [28]. These developments are expanding the scope of peptide therapeutics by accelerating early-stage discovery and enabling more efficient exploration of the vast peptide sequence space [29]. In addition, AI-driven approaches are increasingly being applied to the design and optimization of delivery-related peptides, including CPPs. Despite these technological advances, peptide therapeutics still face significant challenges in achieving optimal therapeutic efficacy, largely due to cell type heterogeneity, limited intracellular delivery, and suboptimal pharmacological performance [1,30,31].

Recent reviews have highlighted the application of AI in peptide design and CPP-based delivery [32,33,34]. However, these studies primarily focus on individual components, such as generative modeling or delivery prediction, without fully addressing how these approaches can be integrated into a unified framework for therapeutic development. In this review, we emphasize an integrated framework that links in silico prediction with in vitro and in vivo evaluation through multi-objective classification and experimental validation. We systematically discuss these aspects in the following sections: (*i*) Overview of Peptide Therapeutics, (*ii*) Chemical Modification Strategies to Enhance Peptide Drug Properties, (*iii*) Cell-Penetrating Peptide-Based Drug Delivery Systems, (*iv*) AI-guided peptide drug design and predictive modeling, and (*v*) future perspectives and development trends in peptide drug research.

## 2. Overview of Peptide Therapeutics

### 2.1. Comparison with Small Molecules and Antibodies

The pharmaceutical landscape encompasses therapeutic modalities classified as small molecules, biologics, and emerging categories including nucleic acid therapeutics and cell-based treatments [8,35]. Small molecules (<500 Da) have constituted the foundation of pharmacotherapy, while biologics—including monoclonal antibodies and therapeutic proteins—represent the fastest-growing market segment [6]. Therapeutic peptides (10–50 amino acids, 500–5000 Da) occupy an intermediate position between small molecules and antibodies (~150 kDa), functioning as hormones, neurotransmitters, and protein–protein interaction modulators with high affinity and specificity [36].

Peptides integrate advantages of both small molecules and antibodies while mitigating class-specific limitations. Primarily, the size fundamentally affects pharmacological properties and biodistribution: small molecules access intracellular targets, antibodies remain confined to extracellular targets, and peptides exhibit intermediate membrane permeability with certain sequences facilitating intracellular delivery [37]. Regarding target specificity, antibodies demonstrate exceptional selectivity, small molecules display lower selectivity with off-target interactions, and peptides achieve antibody-comparable selectivity through multiple contact points while retaining structural simplicity [38]. In this context, peptides can offer a unique advantage by combining the target selectivity typically associated with antibodies and the bioavailability of small molecules [39]. For example, icotrokinra (JNJ-77242113), a peptide that selectively targets the interleukin-23 receptor and binds with single-digit picomolar affinity, has demonstrated significant efficacy in phase 3 trials for plaque psoriasis [40,41,42]. These characteristics, combined with modular chemical synthesis, make peptides highly adaptable to diverse therapeutic areas—from oncology and endocrinology to infectious and neurodegenerative diseases.

However, peptides present distinct pharmacokinetic limitations. Antibodies achieve extended half-lives (2–3 weeks) via FcRn-mediated recycling [43], small molecules exhibit abbreviated half-lives (hours) subject to hepatic/renal clearance [8], and peptides undergo proteolytic degradation yielding minute-to-hour half-lives [44]. Chemical modifications including attachment of polyethylene glycol (PEGylation), cyclization, and non-canonical amino acid (NCAA) incorporation enhance peptide stability [1]. Additionally, small molecules permit oral delivery, antibodies necessitate parenteral administration, and peptides exhibit limited oral bioavailability (<1–2%) due to gastrointestinal enzymatic degradation [13,45], though emerging formulation strategies demonstrate potential [8,13]. Finally, manufacturing employs cost-effective chemical synthesis for small molecules, expensive mammalian cell systems for antibodies, and solid-phase synthesis or recombinant expression for peptides at moderate costs [36,46].

In drug development, the strategy between small molecules, peptides, and antibodies is primarily determined by target accessibility, required binding characteristics, and delivery constraints [47]. Small molecules are typically preferred for intracellular targets with well-defined binding pockets, whereas antibodies are advantageous for extracellular targets requiring high specificity and prolonged circulation [47,48]. Peptides are particularly well-suited for targets with more complex binding interfaces that are often challenging for small molecules, and they possess improved tissue penetration compared to antibodies [49]. Therefore, peptide therapeutics constitute a modality integrating antibody-characteristic specificity with enhanced tissue penetration and reduced immunogenicity, demonstrating superior selectivity relative to small molecules with intracellular target engagement capacity [15,50]. These advantages underscore a paradigm shift wherein peptides transition from niche molecules to a rapidly expanding drug class empowered by multidisciplinary innovation [8,36,50].

### 2.2. Challenges in Peptide Therapeutics

#### 2.2.1. Rapid Metabolic Turnover and Short Duration of Action

The primary biological barriers limiting peptide therapeutics are their susceptibility to enzymatic degradation and rapid systemic clearance [2,8]. Upon exposure to the physiological environment, peptides are immediately targeted by ubiquitous proteases present in the systemic circulation and tissues. For example, GLP-1 has a short half-life of 1–2 min due to rapid inactivation by dipeptidyl peptidase IV (DPP-4) [51]. Similarly, somatostatin is degraded by ubiquitous peptidases within 1–3 min, resulting in low circulating levels that restrict its therapeutic efficacy [52]. Moreover, rapid renal clearance further impairs their pharmacokinetic profile. Most peptides have a molecular weight below the glomerular filtration threshold, enabling their efficient elimination from the body [2]. The effects of proteolytic cleavage and renal filtration result in a short duration of action, necessitating frequent high-dose administration.

#### 2.2.2. Physicochemical Instability and Chemical Degradation

Beyond biological degradation and clearance, therapeutic peptides possess inherently more complicated structures than small molecules. Peptides are susceptible to multiple chemical degradation mechanisms; for instance, sulfur-containing amino acids, like methionine and cysteine, are especially vulnerable to oxidation [53], and asparagine deamidation and diketopiperazine formation alter peptide structure [11,54]. Moreover, physical instability poses a significant challenge, as peptides are prone to conformational unfolding and aggregation under stressors such as temperature or shear [2]. When the formulation pH approaches the isoelectric point, the minimization of net charge leads to precipitation or non-specific adsorption to surfaces, further reducing the effective concentration of the therapeutics.

#### 2.2.3. Immunogenicity Constraints

Immunogenicity remains a critical safety concern for peptide therapeutics, as even peptides derived from endogenous sequences can elicit unwanted immune responses [55,56]. This biological reaction frequently generates anti-drug antibodies (ADAs), which constitute a significant barrier to clinical application by neutralizing the therapeutic efficacy, accelerating clearance, or inducing hypersensitivity reactions. Recent clinical analyses revealed that tirzepatide, a glucose-dependent insulinotropic polypeptide (GIP)/GLP-1 receptor agonist, induced ADAs in 51.1% of patients during phase 3 trials, which were associated with hypersensitivity and injection-site reactions [57]. Therefore, predicting and minimizing immunogenicity are fundamental prerequisites for the successful design and clinical translation of peptide therapeutics.

## 3. Chemical Modification Strategies to Enhance Peptide Drug Properties

Over 600 peptides are currently in preclinical or clinical development, with more than 80 peptide drugs approved worldwide by 2022 [1], and the number of peptide drugs approved for clinical use is steadily increasing globally [8]. This trend underscores a pivotal shift in drug discovery, driven by enhanced delivery technologies and a diversifying therapeutic pipeline. In this section, we highlight major chemical modification strategies that enhance the stability, bioavailability, and therapeutic performance of peptide drugs.

### 3.1. Chemical Modifications Strategies to Enhance Peptide Drug Properties

**Figure 1:**
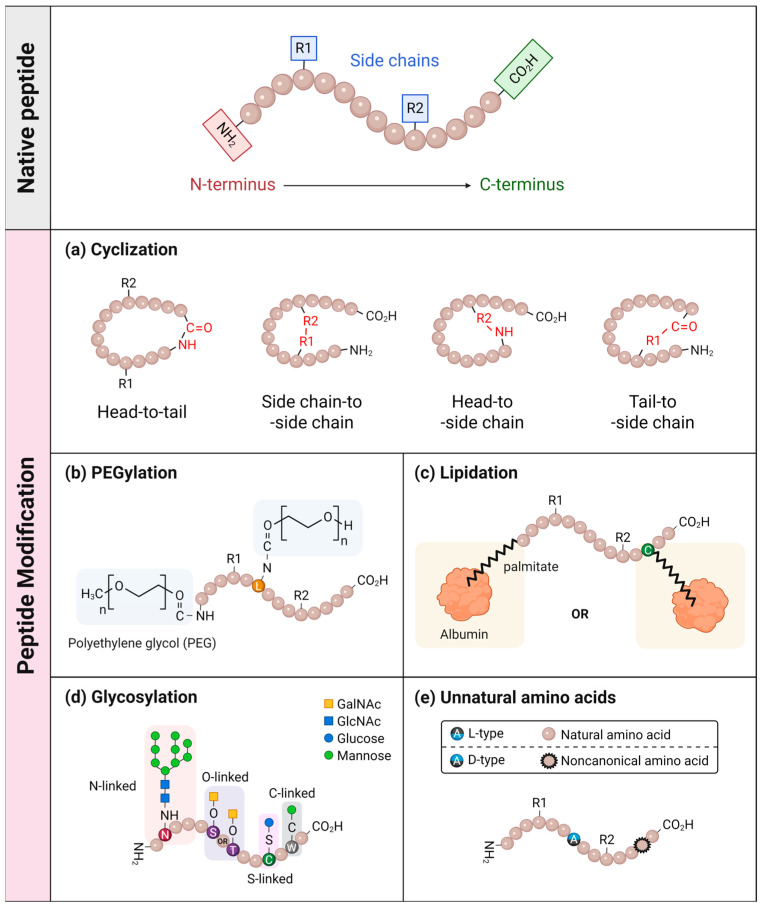
A schematic overview of peptide modification strategies to enhance therapeutic efficacy. The top panel illustrates a native linear peptide with its N-terminus, C-terminus, and side chains (R1, R2). The bottom panels (**a**–**e**) depict various chemical modifications, including cyclization, PEGylation, lipidation, glycosylation, and the incorporation of unnatural amino acids, to improve proteolytic stability and pharmacokinetic profiles. Abbreviations: GalNAc, N-acetylgalactosamine; GlcNAc, N-acetylglucosamine. Created in BioRender. Lee, G. (2026) https://BioRender.com/aeaphcj, accessed on 22 April 2026.

This review systematically surveys chemical modification strategies in peptide drug design, focusing on conformational constraints and conjugation-based approaches, including cyclization, PEGylation, lipidation, and glycosylation. It highlights how these chemical strategies improve peptide stability, target affinity, and drug-like properties and discusses computational design integration and translational challenges. The pharmacological performance of peptides is determined largely by their chemical structure and metabolic resilience. Over the last decade, a wide range of chemical modifications has been introduced to improve stability, enhance binding affinity, and extend half-life (Figure 1, Table 1).

#### 3.1.1. Cyclization

Cyclization is a powerful strategy for overcoming the inherent pharmacological limitations of linear peptides. In their native linear form, most short peptides undergo rapid conformational interconversion due to their high flexibility, resulting in a significant entropic penalty during target binding [58]. Furthermore, the exposed amide bonds in linear sequences are highly susceptible to proteolytic cleavage by peptidases, leading to a short half-life in vivo [59]. To address these challenges, cyclization promotes the stable formation of secondary structures, such as loop or turn structures, thereby increasing both proteolytic stability and cell permeability [1]. By pre-organizing the internal connectivity in the peptide, this structural locking not only shields the backbone against solvent-mediated degradation but also facilitates the effective engagement with target binding sites [58,60]. Direct comparison of linear, monocyclic, and bicyclic peptides in human plasma revealed that proteolytic degradation was restricted exclusively to residues outside macrocyclic ring structures, with a bicyclic peptide (B5) in which nearly all residues were enclosed within the rings retaining full integrity over 24 h [61]. Similarly, cyclic peptides identified through combinatorial screening against thrombin withstood 8 h incubation in simulated gastrointestinal fluids and achieved oral bioavailability of up to 18% in rats—an endpoint that is essentially unattainable for unmodified linear counterparts [62].

Accordingly, a diverse set of techniques has been developed, including head-to-tail cyclization via the linkage between the N- and C-termini, side chain-to-side chain or side chain-to-backbone cyclization (Figure 1a). Head-to-tail cyclization is achieved by covalently linking the N-terminal and C-terminal residues through an amide bond, generating a closed cyclic structure. This elimination of terminal residues increases resistance to exopeptidases, which primarily recognize both N- and C-terminal groups, and simultaneously enhances structural rigidity, thereby limiting the access of endopeptidases to the peptide backbone [63]. Cyclosporine A and gramicidin S are representative clinically approved examples of this strategy [63].

In addition, side chain-to-side chain or side chain-to-backbone cyclization enables macrocycle formation by introducing new covalent bonds—such as disulfide, amide, thioether, triazole, or lactam bridges—either between functional side chains or a side chain and the backbone [64]. Unlike head-to-tail cyclization, these local cross-linking strategies can preserve at least one terminus or both, leaving the terminal functional groups intact, thereby contributing to target recognition [65]. Such selective constraint effectively pre-organizes the peptide into its bioactive conformation. Notably, hydrocarbon stapling is a specialized form of side-chain cyclization that stabilizes α-helices via ruthenium-catalyzed ring-closing metathesis [66]. Additionally, hydrocarbon-stapled peptides exhibit enhanced membrane penetration relative to unstapled sequences, although improvement magnitude varies with ring geometry, backbone amide occlusion, and scaffold type [67,68]. The stapled BH3 peptide developed by Walensky et al. exhibited significant improvements in protease resistance and cellular uptake [69], highlighting the importance of conformational stabilization and pre-organization as key strategies in peptide drug design and modulation of protein–protein interactions. Furthermore, recent advances further highlight the continued relevance of cyclization strategies in peptide drug development, with cyclic and stapled peptides increasingly being explored for improved stability, target affinity, and intracellular delivery [62,70,71,72].

Despite these structural advantages, cyclization also has inherent limitations, such as low yields, and synthetic complexity [73]. In cases where constrained structure fails to pre-organize the peptide into a favorable bioactive conformation, cyclization can impose higher binding enthalpy and lower potency [74]. Therefore, while cyclization is a useful method to design and enhance the efficacy of peptide drugs, its application requires a delicate design to balance these benefits against potential trade-offs.

#### 3.1.2. PEGylation

While cyclization focuses on restricting conformational flexibility to enhance the stability and binding affinity, other strategies improve pharmacological performance by modifying the peptide’s physicochemical properties or leveraging serum components for extended circulation. One of the most successful strategies is PEGylation to peptides [75] (Figure 1b). PEG is composed of repeating ethylene oxide units and possesses favorable properties, including non-biodegradability, minimal toxicity, and low immunogenicity [1]. Conjugation of PEG to peptides increases the hydrodynamic size of the peptides, protecting peptides from proteases by steric hindrance [76]. In addition, the increased size can also reduce renal filtration and prolong the systemic retention of PEGylated peptides [77,78,79]. Indeed, pegvisomant, a modified human growth hormone (GH) at the third α-helix widely used as a GH receptor antagonist, exhibited an extended half-life and low immunogenicity following PEGylation and has been approved for the treatment of acromegaly [80,81].

Despite these advantages, PEGylation can produce polydisperse conjugates due to its heterogeneity in PEG chain length, potentially leading to variable biological effects [82]. Additionally, reduced kidney excretion due to PEGylation has been reported to lead to accumulation in tissues, raising concerns related to macromolecular syndrome, underscoring the importance of optimizing molecular weight and linker chemistry for PEGylation [75]. Nevertheless, PEGylation has been considered a key strategy in peptide drug design, notably for applications where enhanced pharmacokinetic characteristics are required.

#### 3.1.3. Lipidation

Lipidation involves the conjugation of fatty acids, such as palmitic and myristic acids, to peptide residues, promoting reversible binding to serum albumin and extending the half-life [83] (Figure 1c). Lipidated peptides have gained attention as therapeutic agents due to their tunable lipophilicity and enhanced bioavailability [84]. Kurtzhals et al. pioneered this approach by demonstrating that attachment of a fatty acid to a lysine or other residue of a peptide via a linker can facilitates binding to serum albumin, thereby reducing enzymatic degradation and renal clearance [85]. Albumin is the most abundant protein in the blood, with a long half-life of approximately 20 days, and it can serve as a carrier by binding to various drugs [86]. Specifically, fatty acids ranging from C10 to C18 were found to bind to seven sites on albumin [87]. This provides a distinct benefit for lipidated peptides, which maintain a high probability of interaction with albumin and exert their therapeutic effect upon dissociation from albumin [86].

On this basis, the most representative therapeutic peptide developed via lipidation is liraglutide. Liraglutide is a human GLP-1 analog coupled with C16 fatty acid to a lysine at position 26 and extensively used for type 2 diabetes therapy [88]. This approach enabled the extension of the half-life of liraglutide from 1–2 min in its native form to more than 10 h [88]. Furthermore, recent studies have reported that GLP-1 derivatives, including liraglutide and semaglutide, can self-assemble and form spherical micelles or oligomerized states, which are advantageous to sustain the half-life [89]. However, this strategy also requires careful optimization. Lipidation also presents certain limitations, such as a potential trade-off between albumin affinity and receptor potency [90]. Consequently, optimizing parameters, such as acyl chain length and linker design [90], is essential for developing long-acting, stable, and potent peptide therapeutics.

#### 3.1.4. Glycosylation

Glycosylation is another chemical modification strategy used to improve peptide stability, solubility, and half-life [91,92]. Based on the atom of amino acid side chain to which the carbohydrate chain is conjugated, glycosylation can be categorized into four subtypes: N-, O-, C-, or S-glycosylation [93] (Figure 1d). By attaching these glycosyl units to peptides, various pharmacological properties, such as in vivo distribution, cell penetration, metabolic stability, receptor binding, and degradation rate, are affected [91,94,95]. One of the most critical advantages of glycosylation is its ability to improve the aqueous solubility of peptides by masking hydrophobic peptide surfaces with hydrophilic glycans [96,97]. This modification effectively prevents glycosylated peptides from irreversible aggregation and precipitation, which are major obstacles in peptide design [98]. The efficacy of this strategy is well demonstrated by the development of glycosylated somatostatin analogs, featuring a human complex type N-glycan [97]. Ochiai et al. demonstrated that site-specific introduction of glycan moieties not only improved the solubility of the peptide but also extended its half-life and maintained its pharmacological activities without compromising receptor affinity [97]. This example underscores the importance of rational glycan engineering in peptide drug design. However, the tight control over glycan structure, conjugation site, and heterogeneity of glycoform remains challenging [93,94], necessitating careful engineering.

#### 3.1.5. D-Amino Acids and Non-Canonical Residues

Conventional strategies, including cyclization, PEGylation, lipidation, and glycosylation, primarily aim to construct a steric hindrance against the proteolytic activities. In contrast, substituting natural residues with D-amino acids or non-canonical residues fundamentally alters the backbone or side chains of a peptide, rendering it unrecognizable to endogenous enzymes [99] (Figure 1e). Although peptides and proteins are predominantly composed of L-amino acids, D-amino acids—the enantiomers of L-amino acids—occur naturally in certain peptides [100]. The concept of introducing D-amino acids was inspired by the discovery of their presence in bacterial cell walls and non-ribosomal antibiotic peptides, which provide resistance against various peptidases [101]. This natural strategy of enzymatic evasion was subsequently applied to human therapeutics, as exemplified by the development of desmopressin. In this case, N-terminal deamination combined with the substitution of L-arginine by D-arginine at position 8 significantly improved its stability and extended its half-life by preventing rapid degradation [102]. Notably, this D-amino acid strategy has been further advanced, enabling the synthesis of peptides composed entirely of D-amino acids [103]. Liu et al. applied mirror-image phage display technique to synthesize a peptide composed of D-amino acids and identified a D-peptide inhibitor of the p53-MDM2 interaction, which demonstrated proteolytic resistance and nanomolar affinity for its target [103]. These findings underscore the effectiveness of D-amino acid substitution in peptide therapeutics through precise stereochemical control. As a representative clinically approved example of this strategy, octreotide, a synthetic somatostatin analog containing a D-tryptophan residue, has been widely used clinically owing to its enhanced stability and superior pharmacological profile [104].

In contrast, NCAAs are defined as any amino acids that are not part of the 20 standard L-amino acids genetically encoded for ribosomal protein synthesis [105]. Unlike other methods, NCAA introduction can modulate backbone geometry, amide bond properties, and local conformational flexibility, thereby providing advantages for peptide design beyond side-chain substitution alone [106]. For example, as Castro et al. highlighted, α-isobutyric acid (Aib), an α,α-dialkyl glycine, restricts the backbone dihedral angles, thereby promoting the formation of α-helical structures and enhancing peptide stability [106,107]. In addition, similar modulation has been reported for other NCAA subtypes, including proline analogs, β-substituted amino acids, and α,β-dehydroamino acids [106], confirming this approach as effective for designing improved peptide therapeutics.

However, despite their advantages, incorporating NCAAs faces several challenges related to synthetic accessibility and limited translational applicability [108]. While Azam et al. demonstrated that NCAA application can modulate immunogenicity by diminishing T-cell recognition of antigens [109], the overall immunogenic consequences remain complex and poorly understood. Most evidence derives from animal models, and the immunogenic consequences of specific NCAA substitutions may not be directly translatable to human systems. This is because murine and human MHC binding specificities are generally distinct, and certain epitopes are recognized exclusively in humans but not in murine models [110]. In addition, current in silico immunogenicity prediction tools are largely optimized for canonical amino acids and are not inherently designed to accommodate sequences containing D-amino acids and NCAAs, further complicating translational assessments [111].

Collectively, the chemical modification strategies discussed in this section have substantially expanded peptide drug development by improving stability, pharmacokinetics, and binding affinity. However, despite these advances, efficient intracellular delivery remains a major challenge for many peptide therapeutics due to their limited membrane permeability and susceptibility to degradation. To address these limitations, CPPs have emerged as a promising strategy for enhancing the cellular uptake and intracellular delivery of therapeutic cargos. The following section therefore provides an overview of CPP-based drug delivery systems and their therapeutic applications.

## 4. Cell-Penetrating Peptide-Based Drug Delivery Systems

Efficient intracellular and tissue-specific delivery remains a central challenge in the clinical translation of peptide therapeutics. Delivery systems developed to address this challenge can be broadly categorized into peptide-based carriers, such as CPPs, and non-peptidic systems, including exosomes, nanoparticles, and physical delivery methods. In this context, CPPs are distinguished by their short peptide sequences that inherently facilitate cellular uptake, whereas other delivery systems typically rely on vesicular transport, encapsulation, or external physical forces to achieve intracellular delivery [112,113,114]. Building on this distinction, CPPs have gained considerable attention as delivery vehicles capable of facilitating membrane translocation and overcoming biological barriers. Beyond general intracellular delivery, CPP-based systems are being actively explored for targeting complex anatomical sites such as the central nervous system and the pulmonary epithelium [115,116]. In parallel, advances in artificial intelligence are accelerating the identification and optimization of CPP sequences, further expanding their translational potential.

### 4.1. Cell-Penetrating Peptides

Over the past three decades, CPPs, also known as peptide transduction domains (PTDs), have evolved from a biological observation into a broadly applicable intracellular delivery vehicle. The field was initiated by findings that certain transcription factors can cross cell membranes [117], culminating in the 1988 report by Frankel and Pabo showing that the HIV-1 Tat protein enters cells and accumulates in the nucleus [118]. A short arginine-rich region within Tat (residues 47–57), commonly referred to as the TAT peptide, mediates cellular internalization and has become one of the most extensively studied CPPs [119]. These and subsequent discoveries established that short peptide motifs are sufficient to promote cellular uptake [120] and opened the way to using CPPs to deliver therapeutic cargo into selected cells [112,113,121]. Simultaneously, the growing pipeline of protein-based therapeutics—including monoclonal antibodies, enzymes, cytokines, and vaccines—has highlighted both the therapeutic potential of these agents and the practical difficulties associated with their delivery [122,123]. Most approved protein drugs are designed for extracellular targets [124] because the lipophilic plasma membrane forms an effective barrier that prevents large hydrophilic macromolecules from reaching intracellular sites of action [125]. To overcome this limitation, a variety of physical and carrier-based approaches have been explored, including ultrasonic nebulization [126], electroporation [127], calcium phosphate co-precipitation [128], exosomes [129], and nanoparticle systems [114,130]. However, these approaches often come with practical drawbacks, including procedure-related safety issues, technical complexity, and limited control over the final dose and in vivo distribution of the delivered proteins [131,132]. In this context, CPPs have emerged as attractive peptide-based carriers with generally lower immunogenicity and more predictable dosing than many alternative intracellular delivery technologies [133].

CPPs can transport a broad spectrum of bioactive cargos into cells, including proteins [134,135,136,137], peptides [138], DNAs [139], siRNAs [140,141,142,143], and small-molecule drugs [144,145,146]. By promoting the accumulation of these therapeutics in tissues and cellular compartments that are otherwise difficult to reach, CPPs can substantially enhance local drug concentration and treatment efficacy [147,148]. As a result, CPPs are being actively developed and refined as drug delivery vehicles [149] for antimicrobials [150,151,152,153], anti-inflammatory agents [154,155], antineoplastic drugs [156,157,158], other classes of medicines, and neuroprotective compounds [159].

In addition to their role as delivery vectors [160], CPP-based systems can be further enhanced through chemical modification strategies. Modifications such as cyclization, lipidation, and the incorporation of NCAAs can improve proteolytic stability, membrane interaction, and intracellular persistence, thereby complementing CPP-mediated delivery [161]. For example, cyclization of arginine-rich CPPs such as oligoarginine (R8) enhances cellular uptake and proteolytic stability [162,163], while lipidation of CPPs has been shown to increase membrane affinity and facilitate cellular internalization [164,165]. Building on these advances, CPPs are increasingly being integrated with more complex peptide-based delivery platforms. In particular, peptide-based nanoparticles and self-assembling peptide systems have emerged as versatile approaches to improve drug stability, enable controlled or stimuli-responsive release, and enhance intracellular targeting [166,167,168]. These systems function as delivery platforms that can encapsulate therapeutic cargo and protect it from degradation, thereby complementing the primarily membrane-translocating role of CPPs [169]. Furthermore, these platforms can be combined, for example, by coupling efficient cellular uptake with improved pharmacokinetic control, as in CPP-functionalized nanocarriers [170]. Such integrative approaches further expand the applicability of CPP-based systems, including their potential to overcome biological barriers such as the blood–brain barrier.

### 4.2. CPP-Based Drug Delivery to the Central Nervous System

With the rising prevalence of age-related diseases [171,172], there has been a significant surge in research focusing on peptide-based therapeutics, leveraging their inherent biocompatibility [173,174]. The brain is protected from the systemic circulation by several specialized barriers that tightly regulate the movement of molecules between blood, cerebrospinal fluid, and neural tissue [175,176]. Among these, the blood–brain barrier (BBB) is the most restrictive [177]. It is formed by non-fenestrated brain microvascular endothelial cells sealed by complex tight and adherent junctions, supported by a basement membrane, pericytes, and astrocytic end-feet [178]. This neurovascular unit severely limits paracellular diffusion and tightly controls transcellular transport through a repertoire of influx and efflux systems [179,180]. As a result, only a very small subset of systemically administered compounds—typically small (<400 Da), sufficiently lipophilic molecules with limited hydrogen-bonding capacity—can cross the BBB by passive diffusion [181]. Although additional routes such as transporter-, receptor-, or adsorption-mediated transcytosis exist [182], pharmacokinetic data show that most CNS-directed drug candidates fail to reach therapeutic levels in the brain because they cannot effectively traverse this barrier [183,184]. To overcome this obstacle, CPPs and other homing peptides are being explored as alternative delivery platforms to improve drug access to the central nervous system [115,185]. By exploiting their intrinsic ability to interact with cell membranes and undergo transcytosis [186], CPPs can be coupled to small molecules, peptides, proteins, or nanoparticles to facilitate their transport across the BBB [187,188]. In one representative study, systemic administration of a Bcl-xL fusion protein containing the HIV-1 TAT protein transduction domain enabled efficient delivery into neurons in the brain. This approach significantly reduced infarct volume and neuronal apoptosis in a mouse model of focal cerebral ischemia [189]. Accordingly, CPPs offer a strategy to bypass or modulate the barrier, potentially increasing brain exposure to therapeutic agents that would otherwise be excluded and expanding the range of targets that can be pharmacologically addressed within the CNS.

### 4.3. The Pulmonary Drug Delivery System Based on CPPs

The pulmonary drug delivery system represents a highly compelling target for delivery strategies based on CPPs, owing to its direct and non-invasive accessibility via inhalation, its expansive absorptive surface area, and the prevalence of disease mechanisms driven by intracellular signaling [190]. Unlike systemic organs such as the liver, heart, kidneys, and brain which necessitate the crossing of vascular endothelial barriers or the BBB, pulmonary administration allows CPPs to bypass these primary systemic hurdles [191]. Despite these anatomical advantages, the pulmonary epithelium still presents a biological barrier that can limit the efficient intracellular delivery of therapeutic macromolecules. CPPs offer a robust framework for surmounting pulmonary epithelial barriers by promoting active membrane translocation. This enables access to intracellular therapeutic targets that remain largely unreachable for conventional peptide and protein-based medicines [192]. TAT peptide-conjugated siRNA has been shown to be delivered to lung epithelial cells and macrophages following intratracheal administration in mice, resulting in measurable knockdown of target gene expression [193]. In addition, conjugation with TAT significantly enhanced transepithelial transport across the lung epithelium compared with native insulin, demonstrating that CPP conjugation can improve the pulmonary absorption of protein therapeutics [194]. More recently, lung-targeting CPPs such as S7A and R11A have been identified, showing efficient penetration into human bronchial epithelial cells and preferential accumulation in lung tissue in vivo, enabling intracellular delivery of therapeutic siRNA to alveolar epithelial cells [116]. This anatomical advantage facilitates efficient intracellular entry whilst potentially limiting non-specific systemic distribution and associated toxicity, thereby addressing a significant translational bottleneck in CPP-based therapeutics [195,196]. Such capability is particularly pertinent to respiratory pathologies where pathogenic processes are orchestrated by intracellular pathways within epithelial or immune cells. Furthermore, CPPs demonstrate remarkable cargo versatility, supporting the delivery of a diverse array of payloads, including peptides, proteins, nucleic acids, small molecules, and nanocarrier systems. When integrated with inhalation-based delivery, CPP-mediated transport enhances localized drug accumulation within lung tissue while minimizing systemic circulation. This improves the therapeutic index by reducing off-target effects and dose-limiting toxicities [197].

Whilst CPPs often lack inherent cell type specificity and may be internalized by various pulmonary cell populations, this challenge is increasingly being circumvented through sequence optimization and the integration of active targeting moieties [198]. Additionally, pulmonary clearance mechanisms including mucus entrapment, surfactant interactions, and immune surveillance can diminish in vivo delivery efficiency. However, continued advances in formulation science and inhalation technology are effectively mitigating these issues [199]. Finally, the potential for cytotoxicity and immunogenicity during chronic or high-dose inhalation necessitates meticulous CPP design and rigorous safety profiling [200]. These factors should be viewed as manageable hurdles to clinical application rather than fundamental barriers.

However, traditional experimental approaches for identifying therapeutic peptides, primarily reliant on manual modifications and trial-and-error, are inherently time-consuming, costly, and laborious [201]. In this context, the complexity of optimizing peptide sequences and delivery performance highlights the need for more efficient and systematic design approaches. Consequently, AI-based drug design has emerged as a powerful strategy, enabling data-driven peptide sequence generation, predictive modeling of activity and toxicity, and multi-objective optimization, thereby accelerating discovery timelines and improving candidate selection efficiency. In the following sections, we discuss the historical advances and emerging strategies in peptide drug design enabled by artificial intelligence.

## 5. AI-Guided Peptide Drug Design and Predictive Modeling

### 5.1. Evolution of Algorithmic Frameworks for Peptide Drug Development

**Figure 2:**
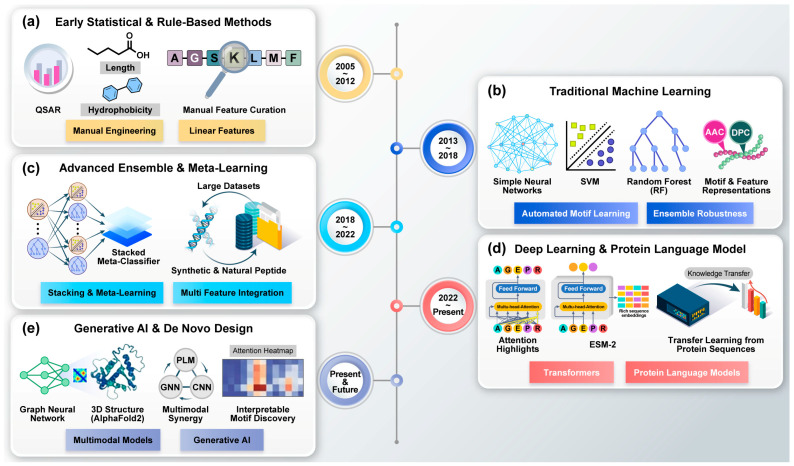
The evolution of algorithmic approaches for peptide drug discovery and design. The timeline illustrates the chronological progression across five distinct stages: (**a**) early statistical and rule-based methods, such as QSAR (2005–2012); (**b**) traditional machine learning applications including SVM and RF (2013–2018); (**c**) advanced ensemble and meta-learning strategies (2018–2022); (**d**) the application of deep learning and Protein Language Models (PLMs) (2022–present); and (**e**) the current and future integration of generative AI for de novo design. Abbreviations: QSAR, Quantitative Structure–Activity Relationship; SVM, Support Vector Machine; RF, Random Forest; PLMs, Protein Language Models; GNN, Graph Neural Network; CNN, Convolutional Neural Network; ESM-2, Evolutionary Scale Modeling 2.

Over the past two decades, the computational landscape for peptide drug discovery has evolved from empirical, physicochemical rule-based approaches to advanced AI frameworks (Figure 2). Early studies relied on statistical correlations between peptide biochemical activity and physicochemical descriptors such as net charge, hydrophobicity, and amphipathicity, forming the basis of quantitative structure–activity relationship (QSAR) models [202]. As peptide datasets expanded, ML approaches—particularly Support Vector Machines (SVMs) and Random Forest (RF) models—became widely adopted, enabling more systematic feature engineering and sequence classification through descriptors such as amino acid composition [203,204,205,206,207,208,209,210]. As peptide datasets grew in complexity, subsequent developments introduced ensemble learning strategies and meta-learning frameworks, which integrated multiple classifiers to improve predictive stability and reduce model bias [211,212,213,214]. More recently, transformer-based protein language models (PLMs) [215], including models such as ProtBERT, ProtT5 [29], and ESM [216], have significantly advanced peptide prediction by generating high-dimensional sequence embeddings that capture structural and evolutionary information without manual feature engineering [216,217,218]. This progression—from empirical descriptors to deep representation learning—has substantially improved the accuracy and scalability of peptide activity prediction and rational design [219]. Nevertheless, these models are not without limitations: their computational demands can be substantial, and the high-dimensional embedding spaces they produce often resist straightforward biological interpretation, presenting a trade-off between predictive power and mechanistic insight.

In parallel, AI-driven predictive modeling frameworks have continued to expand the scope of computational peptide discovery by integrating large-scale biological data with advanced learning algorithms. Increasingly, generative AI approaches—including diffusion-based models and structure-aware sequence design frameworks—are being applied to enable the de novo design of peptides with tailored therapeutic properties [220,221]. Landmark advancements, such as the work by Cao et al., have demonstrated the ability to design high-affinity binders from target structures alone [222]. Furthermore, generative models are now being expanded to include non-canonical amino acids and chemically modified residues, essential for enhancing the proteolytic stability and drug-likeness of next-generation peptide therapeutics [223]. Despite this progress, several challenges remain. Many computationally designed sequences fail experimental validation due to aggregation, poor solubility, or off-target effects [224,225]. In addition, current generative models still struggle to simultaneously optimize across multiple pharmacological objectives such as potency, selectivity, and metabolic stability.

### 5.2. Artificial Intelligence Approaches to CPP Prediction

**Figure 3:**
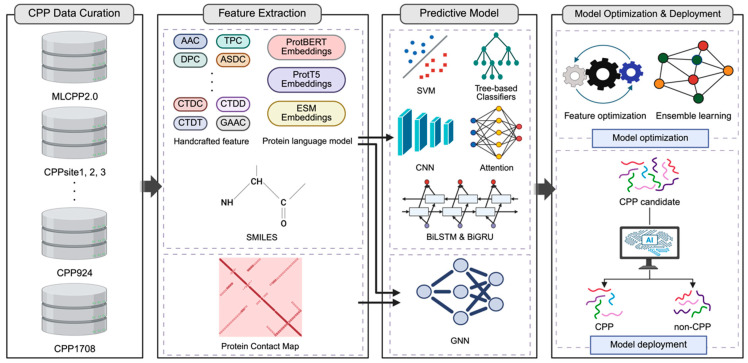
Primary stages in the development of a CPP prediction model. The pipeline consists of four primary stages. CPP Data Curation: Benchmarking diverse datasets, including MLCPP2.0, CPPsite1, CPPsite2, CPPsite3, CPP924, and CPP1708, to ensure data robustness. Feature Extraction: Extracting multi-modal information through handcrafted features, PLMs, SMILES sequences, and protein contact maps. Predictive Model Construction: Building a hybrid architecture that integrates traditional machine learning (SVM, tree-based classifiers) and deep learning frameworks (CNN, BiLSTM/BiGRU, Attention) using handcrafted and PLM-based features. GNN is constructed by incorporating protein contact maps with existing features for enhanced prediction. Model Optimization & Deployment: Refining model performance via hyperparameter optimization and ensemble learning techniques, followed by the deployment of the finalized predictive model. Abbreviations: AAC, Amino Acid Composition; TPC, Tripeptide Composition; DPC, Dipeptide Composition; ASDC, Adaptive Skip Dipeptide Composition; CTDC, Composition of k-Spaced Amino Acid Pairs (Composition); CTDD, Composition, Transition, Distribution (Distribution); CTDT, Composition, Transition, Distribution (Transition); GAAC, Grouped Amino Acid Composition; ProtBERT, Protein Bidirectional Encoder Representations from Transformers; ProtT5, Protein Text-to-Text Transfer Transformer; ESM, Evolutionary Scale Modeling; SMILES, Simplified Molecular Input Line Entry System; SVM, Support Vector Machine; CNN, Convolutional Neural Network; BiLSTM, Bidirectional Long Short-Term Memory; BiGRU, Bidirectional Gated Recurrent Unit; GNN, Graph Neural Network; CPP, Cell-Penetrating Peptide. Created in BioRender. Lee, G. (2026) https://BioRender.com/8ayeots, accessed on 22 April 2026.

As a representative example of these advances, the prediction of CPPs has greatly benefited from AI-driven modeling approaches (Figure 3), and representative CPP prediction models are summarized in Table 2. The development of these models typically begins with the curation of high-quality benchmark datasets, such as MLCPP2.0 [226], CPPsite1 [227], CPPsite2 [228], CPPsite3 [229], CPP924 [230], and CPP1708 [231], which provide experimentally validated peptide sequences for training and evaluation. Details of these datasets, including dataset size, sequence diversity (e.g., redundancy reduction using CD-HIT or duplicate removal) [114,232], and the level of experimental validation, are summarized in Table 3. Once curated, peptide sequences must be converted into computationally processable representations. This is achieved through a range of encoding strategies, including handcrafted descriptors capturing physicochemical properties (e.g., hydrophobicity, charge, and amino acid composition) [233], SMILES-based [234] molecular representations processed through cheminformatics libraries such as RDKit [235] or DeepChem [236], and PLM embeddings derived from large-scale pretrained models such as ProtBERT, ProtT5, and ESM [29,216]. In addition, structural representations such as protein contact maps can be used to capture spatial relationships between amino acids and account for three-dimensional folding patterns [216,237].

These diverse representations are analyzed through a wide range of ML and DL architectures. Traditional ML approaches, including SVM and tree-based classifiers, remain widely used due to their robustness and interpretability when working with structured features [17,207,208,209,211,226,230,238,239,240,241,242,246,247]. More recently, DL architectures have enabled more sophisticated modeling of peptide sequences. Convolutional neural networks (CNNs) identify local sequence patterns [243,248], whereas recurrent neural networks such as bidirectional long short-term memory (BiLSTM) and bidirectional gated recurrent units (BiGRUs) capture long-range dependencies within peptide sequences. Attention-based mechanisms further enhance predictive performance by highlighting functionally important residues [29,244,245]. In addition, graph neural networks (GNNs) integrate structural information derived from protein contact maps, allowing models to capture complex three-dimensional relationships between amino acids [231]. These feature representations and model architectures are often combined within integrated predictive frameworks that employ hyperparameter optimization and ensemble learning strategies to improve model generalization [246]. To ensure accessibility and reproducibility within the scientific community, many of these state-of-the-art models are deployed as open-source tools or user-friendly web servers, bridging the gap between computational development and experimental application.

### 5.3. Computational Frameworks for AI-Driven Peptide Drug Discovery

**Figure 4:**
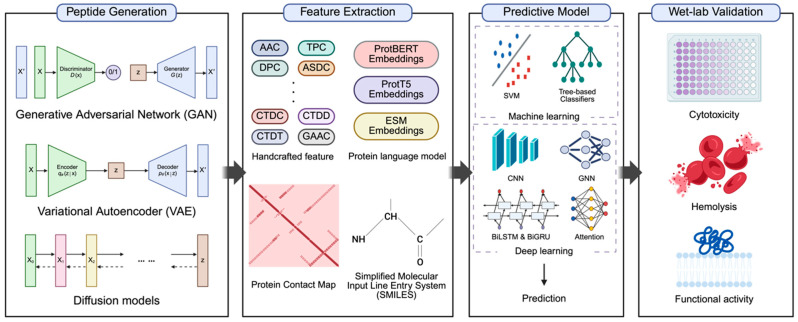
AI-driven workflow for peptide drug discovery and experimental validation. The schematic illustrates the comprehensive workflow for AI-driven peptide discovery and validation. Peptide Generation: Diverse candidate sequences are generated using generative AI architectures, including GAN, VAE, and Diffusion models. Feature Extraction: Generated sequences are transformed into computationally processable formats through handcrafted features, PLM embeddings, protein contact maps, and SMILES-based descriptors. Predictive Model: The extracted features are analyzed using ML and DL architectures. ML utilizes models such as SVM and tree-based classifiers, while DL employs architectures including CNN, BiLSTM/BiGRU, Attention-based mechanisms, and GNNs. Wet-Lab Validation: Predicted peptides are experimentally validated through various assays—such as cytotoxicity and hemolysis tests—to ensure safety and functional activity assays to verify therapeutic efficacy. Abbreviations: GAN, Generative Adversarial Network; VAE, Variational Autoencoder; PLM, Protein Language Model; SMILES, Simplified Molecular Input Line Entry System; ML, Machine Learning; DL, Deep Learning; SVM, Support Vector Machine; CNN, Convolutional Neural Network; BiLSTM, Bidirectional Long Short-Term Memory; BiGRU, Bidirectional Gated Recurrent Unit; GNNs, Graph Neural Networks. Created in BioRender. Lee, G. (2026) https://BioRender.com/v6s6jmq, accessed on 22 April 2026.

With the rapid advancement of ML and DL, it is possible to design peptide drugs by considering a diverse array of complex factors [19,20,21]. To accurately predict the efficacy of designed peptides, diverse computational representations and feature extraction strategies have been developed, enabling complex molecular and biological information to be encoded into machine-readable formats [29,215,233,234]. These representations, together with modern ML and DL architectures, allow for rapid in silico screening of candidate sequences prior to chemical synthesis, thereby overcoming many practical limitations of traditional experimental approaches [249]. Together, these approaches establish an integrated pipeline from sequence generation to functional evaluation (Figure 4).

Despite these sophisticated computational breakthroughs, rigorous wet-lab validation—including functional assays and safety profiles—remains indispensable to ultimately confirm the biological activity and safety of the predicted candidates. Such validation typically involves functional activity assays to verify therapeutic efficacy, as well as safety assessments including cytotoxicity and hemolysis tests [250]. The ability to design a vast number of peptide candidates with minimal cost and rapid screening provides a powerful advantage that complements traditional experimental verification. Importantly, CPP-mediated delivery should not be interpreted solely at the peptide sequence level, but rather as a complex system involving CPP–cargo assemblies (e.g., nucleic acids, proteins, or small molecules) [225,251]. In such systems, physicochemical properties including particle size, surface charge, and structural flexibility critically influence cellular uptake depending on the target cell type and biological barrier [113,114]. Therefore, current AI models show strong potential for designing optimized CPP sequences, while further research on CPP-mediated delivery systems remains necessary. In this regard, AI approaches provide a meaningful, albeit partial, contribution to peptide therapeutic development. To support these advances, continued improvements in computational algorithms and AI architectures will be essential.

## 6. Future Perspectives and Development Trends

The peptide therapeutics field stands at a transformative juncture, propelled by convergent technological innovations that promise to overcome longstanding pharmacokinetic and delivery limitations [36,252]. AI algorithms are revolutionizing peptide discovery through deep generative models, enabling rapid de novo design of optimized sequences with predicted bioactivity, reduced toxicity, and enhanced membrane permeability [28,253]. Integration of multi-omics platforms, including immunopeptidomics and proteomics, facilitates comprehensive neoantigen identification for personalized cancer vaccines, with clinical trials demonstrating robust T-cell responses and sustained progression-free survival in melanomas, glioblastomas, and other malignancies [254,255,256].

Despite these advances, several critical challenges remain in translating these innovations into effective peptide delivery strategies. The development of cargo-specific design strategies will be essential, as delivery efficiency varies significantly among proteins, nucleic acids, and small molecules. In this context, future AI frameworks should move beyond sequence-based design and incorporate delivery-relevant parameters, including CPP–cargo interactions, particle size, and surface charge, to enable more accurate prediction of cellular uptake across diverse biological barriers [114].

The use of peptide generation models to design candidate sequences, followed by multi-objective optimization and evaluation using predictive models (e.g., classification, hemolysis, and minimum inhibitory concentration prediction), enables efficient assessment of potential peptide therapeutics and can partially replace early-stage experimental screening [257]. However, such approaches cannot fully substitute for comprehensive wet-lab validation and clinical evaluation. Accordingly, the next phase of peptide therapeutics development is expected to be driven by the integration of AI-enabled sequence design and predictive modeling with wet-lab validation and clinical evaluation of targeted peptide delivery to disease-relevant tissues.

These advances converge to position peptide therapeutics as precision medicine agents capable of targeting previously undruggable intracellular pathways. This can be achieved by improving delivery efficiency and reducing toxicity, thereby expanding the therapeutic window while minimizing off-target effects. Furthermore, this convergence heralds a new era in which computational design and experimental validation synergistically accelerate clinical translation, provided that coordinated advances are achieved in model interpretability, robust experimental validation pipelines, and regulatory frameworks for AI-designed therapeutics.

## 7. Conclusions

Peptide therapeutics are increasingly recognized as an important drug modality bridging small molecules and biologics, combining high target specificity with generally favorable safety profiles. However, their clinical translation remains constrained by limited stability, rapid clearance, and low bioavailability. This review highlights how rational chemical modifications, including cyclization, PEGylation, lipidation, and the incorporation of non-canonical residues, improve proteolytic resistance and pharmacokinetic performance. In parallel, delivery strategies based on CPPs expand intracellular and tissue-selective targeting. Importantly, the integration of AI represents a substantive shift in the field. Generative models support de novo sequence design, whereas ML and DL enable multi-parameter optimization and candidate prioritization, thereby streamlining the discovery pipeline and reducing experimental attrition. The integration of AI-driven peptide design with multi-parameter optimization and advanced delivery strategies is expected to enable access to previously challenging therapeutic targets. Although challenges related to manufacturability and immunogenicity remain, advances in CPP-based delivery strategies, as one of several approaches to peptide delivery, alongside other strategies such as nanoparticles, exosomes, and peptide–drug conjugates, position peptide therapeutics as a key component of future precision pharmacotherapy.

In this context, AI enables rapid peptide design and evaluation through the integration of generative models and multi-objective optimization, thereby accelerating the identification of promising therapeutic candidates. Nevertheless, comprehensive wet-lab validation and clinical evaluation remain essential, highlighting the need for integrated platforms that combine computational design with experimental validation.

## Figures and Tables

**Table 1:** Comparative overview of chemical modification strategies in therapeutic peptide drug design.

Strategy	Mechanism	Key Advantages	Key Limitations
Cyclization (head-to-tail)	Covalent N-to-C terminal linkage; eliminates free termini	Strong exopeptidase resistance; enhanced structural rigidity; full plasma integrity over 24 h demonstrated for bicyclic formats	Reduced binding affinity if bioactive conformation not preserved; synthetic complexity; low yields
Cyclization (side chain/stapling)	Intramolecular cross-links via disulfide, lactam, thioether, triazole, or hydrocarbon bridges; α-helix stabilization via RCM	Preserves termini for target recognition; pre-organizes bioactive conformation; variable improvements in cell permeability	Permeability gains are scaffold- and geometry-dependent; not universally predictable; ruthenium catalysis adds synthetic burden
PEGylation	Covalent conjugation of polyethylene glycol; increases hydrodynamic radius	Reduced renal clearance; steric protection from proteases; low immunogenicity	Polydisperse conjugates; variable biological effects; tissue accumulation risk (macromolecular syndrome)
Lipidation	Fatty acid conjugation promoting reversible albumin binding	Extended half-life via albumin recycling (~20-day albumin t½); tunable lipophilicity; enhanced bioavailability	Trade-off between albumin affinity and receptor potency; requires optimization of acyl chain length and linker
Glycosylation	Attachment of glycan units (N-, O-, C-, or S-linked) to amino acid side chains	Improved aqueous solubility; prevents aggregation and precipitation; extended half-life; maintained receptor affinity	Heterogeneity of glycoforms; tight control over conjugation site and glycan structure is technically challenging
D-Amino acid substitution	Replacement of L- with D-amino acid residues; renders backbone unrecognizable to endogenous proteases	Fundamental enzymatic evasion; proteolytic resistance; nanomolar target affinity achievable; full D-peptide synthesis possible	Altered pharmacodynamics; potential immunogenic considerations; synthetic cost of D-residues
Non-canonical amino acids (NCAAs)	Modulation of backbone geometry, dihedral angles, and amide bond properties beyond side-chain substitution	Promotes defined secondary structures (e.g., α-helix via Aib); enhanced stability; broad structural tunability	Low translational efficiency; synthetic accessibility challenges; potential reduction in T cell antigen recognition

**Table 2:** List of AI tools for CPP prediction.

Predictor	Classifier, Years	Dataset Size	Feature Encodings	Evaluation Strategy	Accuracy (Validation/ Independent)	Reference
CellPPD	SVM, 2013	708/708 99/99	AAC, DPC, and BPF	5-fold CV	0.974/0.813	[207]
C2Pred	SVM, 2016	411/411 111/34	DPC-based	10-fold CV	0.836/0.924	[208]
SkipCPP-Pred	RF, 2017	462/462	Adaptive k-skip-2-g	LOOCV	0.906/–	[230]
CPPred-RF	RF, 2017	462/462	PseAAC, ASDC, and PCP	LOOCV	0.916/–	[209]
CPPred-FL	RF, 2018	462/462	Compositional information, Sequence information, and Position information	10-fold CV	0.921/–	[238]
MLCPP	ERT, 2018	427/427 311/311	AAC and PCP	10-fold CV	0.883/0.896	[17]
KELM-CPPpred	KELM	408/408 96/96	ACC, DPC, PseAAC, and motif-based hybrid features	10-fold CV	0.862/0.831	[239]
PEPred-Suite	RF, 2019	370/370 92/92	Sequence-based features	10-fold CV	AUC: 0.952/0.878	[240]
TargetCPP	GB, 2020	462/462 111/34	CPSR, CTD, SAAC, and ITF	LOOCV	0.935/0.882	[241]
StackCPPred	XGB, LGB, SVM, KNN, and RF, 2020	462/462	RECM-Composition, RECM-DWT, and PseRECM	LOOCV	0.945/–	[211]
BChemRF-CPPred	ANN, SVM, and GPC, 2021	300/300 75/75	AAC, DPC, PseAAC, and PCP	10-fold CV	0.876/0.906	[242]
Pep-CNN	imCNN	370/370 92/92	Sequence-based features	10-fold CV	AUC: 0.993/0.988	[243]
MLCPP2.0 (Layer1)	SVM, RF, AB, LGB, GB, XGB, and ERT, 2022	573/573 157/2184	AAC, DPC, TPC, CKSAAGP, and PCP	10-fold CV	0.913/0.934	[226]
SiameseCPP	Siamese neural network + Contrastive Learning, 2023	462/454 573/2184	ProtBERT, One Hot Encoding, and Transformer + BiGRU encoder	8:2 Split	0.961/0.959	[244]
PractiCPP	MLP, 2024	462/462 649/649,000	Transformer encoder, Morgan Fingerprint, and ESM2 embedding	10-fold CV	0.956/–	[245]
CPPpred-En	CB, ERT, and GB, 2025	462/454 573/2184	TPC, CTDC, ProtT5_XL_BFD, ESM1v, ESM1b, and ESM2	5-fold CV	0.972/0.961	[246]
GraphCPP	GNN, 2025	Train: 1586 Validation: 122 Test: 121	SMILES, and Node-wise feature embedding	3-fold CV	–/0.795	[231]
PerseuCPP	ERT, 2025	967/967, (157/2170, 462/462, 90/98)	AAC, DPC, TPC, CKSAAGP, and PCP	10-fold CV	–/0.989, 0.970, 0.811	[247]

Abbreviations: AAC, amino acid composition; AB, adaboosting; AI, artificial intelligence; ANN, artificial neural network; ASDC, adaptive skip dipeptide composition; AUC, area under the curve; BiGRU, bidirectional gated recurrent; BPF, binary profile; CB, catboost; CKSAAGP, composition of k-spaced amino acid group pairs; CNN, convolutional neural network; CPP, cell-penetrating peptide; CPSR, composite protein sequence representation; CTD, composition transition and distribution; CTDC, composition transition and distribution–composition; CV, cross-validation; DPC, dipeptide composition; DWT, discrete wavelet transform; ERT, extremely randomized tree; ESM, evolutionary scale modeling; GB, gradient boosting; GNN, Graph Neural Network; GPC, gaussian processes classifier; imCNN, improved convolutional neural network; ITF, information theory feature; KELM, kernel extreme learning machine; KNN, k-nearest neighbor; LGB, light gradient boosting; LOOCV, leave-one-out cross-validation; MLP, multi-layer perceptron; PCP, physicochemical property; ProtBERT, protein bidirectional encoder representations from transformers; ProtT5_XL_BFD, protein text-to-text transfer transformer extra-large pretrained on big fantastic database; PseAAC, pseudo amino acid composition; RECM, residue energy content matrix; RF, random forest; SAAC, split amino acid composition; SMILES, simplified molecular input line entry system; SVM, support vector machine; TPC, tripeptide composition; XGB, extreme gradient boosting.

**Table 3:** Overview of benchmark CPP datasets used for model development.

Dataset	Dataset Size (CPP/Non-CPP)	Sequence Diversity	Experimental Validation	Reference
MLCPP2.0	Train—573/573 Independent—157/2184	CD-HIT 80% (Train) CD-HIT 90% (Independent—CPP) CD-HIT 70% (Independent—non-CPP)	Mixed (Experimentally validated + predicted)	[226]
CPPsite1	741 (CPP only)	Exact Duplicates removed	Experimentally validated	[227]
CPPsite2	1699 (CPP only)	Exact Duplicates removed	Experimentally validated	[228]
CPPsite3	4143 (CPP only)	Exact Duplicates removed	Experimentally validated	[229]
CPP924	462/462	CD-HIT 80% (CPP)	Experimentally validated	[230]
CPP1708	854/854	CD-HIT 70%	Mixed (Experimentally validated + predicted)	[231]

Abbreviations: CPP, Cell-Penetrating Peptide.

## Data Availability

No new data were created or analyzed in this study. Data sharing is not applicable to this article.

## Abbreviations

The following abbreviations are used in this manuscript:

AAC	Amino Acid Composition
ACPs	Anticancer Peptides
ADAs	Anti-Drug Antibodies
AI	Artificial Intelligence
Aib	α-isobutyric acid
AMPs	Antimicrobial Peptides
ASDC	Adaptive Skip Dipeptide Composition
AVPs	Antiviral Peptides
BBB	Blood–Brain Barrier
BPF	Binary Profile
BiGRU	Bidirectional Gated Recurrent Unit
BiLSTM	Bidirectional Long Short-Term Memory
CB	CatBoost
CD47	Cluster of Differentiation 47
CellPPD	Cell-Penetrating Peptide prediction database/tool
CKSAAGP	Composition of k-spaced amino acid group pairs
CNN	Convolutional Neural Network
CNS	Central Nervous System
CPP	Cell-Penetrating Peptide
CPSR	Composite Protein Sequence Representation
CTD	Composition Transition and Distribution
CTDC	Composition Transition and Distribution–Composition
CV	Cross-Validation
DPC	Dipeptide Composition
DL	Deep Learning
DPP-4	Dipeptidyl Peptidase IV
DWT	Discrete Wavelet Transform
ESM	Evolutionary Scale Modeling
ESM-2	Evolutionary Scale Modeling 2
GAAC	Grouped Amino Acid Composition
GalNAc	N-acetylgalactosamine
GANs	Generative Adversarial Networks
GB	Gradient Boosting
GBM	Glioblastoma
GDF15	Growth Differentiation Factor 15
GIP	Glucose-Dependent Insulinotropic Polypeptide
GlcNAc	N-acetylglucosamine
GLP-1	Glucagon-Like Peptide-1
GLP-1RAs	Glucagon-Like Peptide-1 Receptor Agonists
GNN	Graph Neural Network
GPs	Glycoproteins
GH	Growth Hormone
HIV	Human Immunodeficiency Virus
imCNN	Improved Convolutional Neural Network
ITF	Information Theory Features
KELM	Kernel Extreme Learning Machine
KNN	k-Nearest Neighbor
LGB	Light Gradient Boosting
LOOCV	Leave-One-Out Cross-Validation
ML	Machine Learning
MLP	Multi-Layer Perceptron
mRNA	Messenger Ribonucleic Acid
NCAAs	Non-Canonical Amino Acids
NLCs	Nanostructured Lipid Carriers
PD-L1	Programmed Death-Ligand 1
PEI	Polyethylenimine
PEG	Polyethylene Glycol
PEO-PC7A	Amphiphilic block polymer used for pH-responsive delivery
PLGA	Poly(lactide-co-glycolide)
PLM	Protein Language Model
ProtBERT	Protein Bidirectional Encoder Representations from Transformers
ProtT5_XL_BFD	Protein text-to-text transfer transformer extra large pre-trained on Big Fantastic Database
PTDs	Peptide-Transduction Domains
QSAR	Quantitative Structure-Activity Relationship
RECM	Residue Energy Content Matrix
RF	Random Forest
SAAC	Split Amino Acid Composition
SMILES	Simplified Molecular Input Line Entry System
SLNs	Solid Lipid Nanoparticles
SVM	Support Vector Machine
TPC	Tripeptide Composition
VAE	Variational Autoencoder
XGB	Extreme Gradient Boosting
